# Mobility and freedom of movement: A novel out-of-hospital treatment for pediatric patients with terminal cardiac insufficiency and a ventricular assist device

**DOI:** 10.3389/fcvm.2022.1055228

**Published:** 2022-11-16

**Authors:** Kathrin Rottermann, Sven Dittrich, Oliver Dewald, Andreas Teske, Nicola Kwapil, Steffen Bleck, Ariawan Purbojo, Frank Münch

**Affiliations:** ^1^Department of Pediatric Cardiology, University Hospital Erlangen, Friedrich-Alexander-University Erlangen-Nuremberg (FAU), Erlangen, Germany; ^2^Department of Pediatric Cardiac Surgery, University Hospital Erlangen, Friedrich-Alexander-University Erlangen-Nuremberg (FAU), Erlangen, Germany

**Keywords:** home care environment, ventricular assist device (VAD), EXCOR^®^, pediatric, heart failure, tele-medicine

## Abstract

**Background:**

Due to rapid medical and technological progress, more and more pediatric patients with terminal cardiac insufficiency are being implanted with a ventricular assist device as a bridge to transplant without legal approval for hospital discharge. EXCOR^®^ Active is a recently developed mobile driving unit for the EXCOR^®^ ventricular assist device (EXCOR^®^ VAD) with a long-lasting battery life that can manage small blood pumps, offering improved mobility for pediatric patients. This study strives to elaborate the requirements necessary for a safe home healthcare environment (HHE) for pediatric patients on EXCOR^®^ VAD powered by the EXCOR^®^ Active driving unit.

**Materials and methods:**

Patient- and device-related preconditions (medical, ethical, psychological, technical, structural, organizational) were analyzed with regard to feasibility and safety in three individual patient cases. Included were pediatric patients with terminal cardiac insufficiency in a stable medical condition receiving in-hospital treatment with a univentricular or biventricular EXCOR^®^ VAD powered by EXCOR^®^ Active. Analysis was single-center, data was obtained 05/2020–02/2022.

**Results:**

A total of three patients on EXCOR^®^ VAD were identified for HHE treatment with the EXCOR^®^ Active driving unit. Switch was performed safely and increased mobility led to improved psychomotor development and improved quality of life. No complications directly related to HHE-treatment occurred. One patient recently underwent an orthotopic heart transplant, one patient remains in HHE, and one patient died due to a complication not related to the HHE. Ethical approval for off-label use was obtained and patients and parents were given the required technical training and psychological support. Caregivers and medical professionals involved in the patients’ care at home were briefed intensely. Remote consultations were implemented and interdisciplinary in-hospital checks reduced to a long-term 4-week-scheme.

**Conclusion:**

While it is challenging to discharge pediatric patients being treated with a paracorporeal ventricular assist device (EXCOR^®^ VAD) from hospital, it is feasible and can be managed safely with the novel driving unit EXCOR^®^ Active. A HHE may help to improve patients’ psychomotor development, offer normalized social contacts and strengthen both patients’ and parents’ physical and mental resources. Legal approval and another study with a larger sample size are warranted.

## Introduction

As in the majority of countries there are currently no official legal options for discharging pediatric patients with a cardiac support system under the age of 16 years from hospital, the off-label use of adult continuous-flow devices in children has evolved, introducing the possibility of discharge while on VAD support in the pediatric population (1). Only exception to this are the United States, where the Heartmate 3 has received pediatric labeling with no lower limit of age and its implantation solely depends on the anatomic circumstances.

Adolescent patients with a body weight exceeding 45 kg can benefit from the implantation of LVAD systems (left ventricular assist device) especially designed for discharge from hospital into domestic surroundings (Heartmate) (2). Adult patients requiring paracorporeal biventricular devices (BVAD) with a blood pump size of 60 and 80 ml can be discharged with the EXCOR^®^ Mobil by Berlin Heart GmbH (3, 4). So far, the only other option for patients requiring smaller blood pump sizes is in-hospital treatment with the stationary IKUS driving unit (Berlin Heart GmbH) that can be used for all EXCOR^®^ blood pumps (5, 6). Unfortunately, the IKUS has a battery life of only 30 min and thus considerably limits patients’ mobility (7). Focusing on this particular patient group, Berlin Heart GmbH has recently developed a novel electro-pneumatic mobile drive unit called EXCOR^®^ Active (8), that has been especially designed for the operation of the paracorporeal system EXCOR^®^ VAD and can be used for either univentricular assist (LVAD and RVAD, left and right ventricular assist device) or for biventricular assist (BVAD) (6, 9). Both devices, the IKUS and EXCOR^®^ Active, can be used for both pediatric and adult patients with terminal cardiac insufficiency (9).

The use of the EXCOR^®^ VAD in combination with EXCOR^®^ Active as its driving unit has already been proven to be feasible for internal and external usage within the setting of professional health institutions. Unfortunately, this system, which has a guaranteed battery life of approximately 12 h, has not yet been approved for use in a home healthcare environment (HHE) (10).

The objective of this work was to elaborate potential requirements for the possible hospital discharge of pediatric patients with a VAD in combination with a mobile driving unit and a blood pump size smaller than 60 ml.

## Materials and methods

The elaboration of the necessary requirements for the successful use of EXCOR^®^ Active in a HHE was split into medical, ethical, psychological, technical, structural and organizational aspects:

### Medical inclusion criteria for study patients were defined as follows

- Pediatric patient with terminal cardiac insufficiency undergoing in-hospital treatment with a univentricular or biventricular paracorporeal ventricular assist device and a paracorporeal blood pump size authorized for EXCOR^®^ Active.
- Possible hospital discharge due to being in a stable medical condition on current VAD treatment (no current infection, no wound dehiscence, stable laboratory parameters with an emphasis on coagulation, stable echocardiographic findings with respect to ventricular function, ventricular filling, the presence of intracavitary thrombi, and valvular insufficiencies).

### Medical data was analyzed

- By retrospective review of medical records.
- By real-time analysis of echocardiography, ECG and anthropometric and laboratory values.
- By prospective analysis of motor development.

### Technical requirements included

- Definition of VAD-related stable condition under treatment with EXCOR^®^ Active.
- Technical infrastructure for the secure exchange of regular and emergency data and patient-related data/videoconferencing between parents and professionals at any time.

### Ethical considerations related to

- The possible risks and benefits for patients and parents as elaborated among the team and affected parents, and a favorable opinion was given by the independent ethics committee of the Friedrich-Alexander-University of Erlangen (EB 395, 27.05.2020).

### Psychological aspects

- Were investigated by individual interviews with concerned patients/parents during the evaluation process and after successful discharge from hospital.
- Related to the current quality of life were obtained using the Pediatric Quality of Life Inventory™ (PedsQL) Family Impact Module. The PedsQL is a validated and widely used parent self-reporting tool designed to assess the impact of pediatric chronic health conditions on parents and the family. It includes six subscales measuring parents’ self-reported functioning: Physical Functioning (six items), Emotional Functioning (five items), Social Functioning (four items), Cognitive Functioning (five items), Communication (three items), and Worry (five items). There are also two subscales measuring parent-reported family functioning: Daily Activities (three items) and Family Relationships (five items). The Total Score is calculated as the mean score of all eight subscales. Answers vary from 0 (“never”) to 4 (“almost always”) on a 5-point Likert scale. The items are reversed upon scoring and summed on a scale of 0 to 100, with a higher score indicating a better health-related quality of life (HRQL), and a lower score indicating a poorer HRQL (11, 12).
- The HRQL of 2 discharged patients (patient 2 and 3) was individually compared to the HRQL data of hospital-based EXCOR^®^ VAD patients (0.1 to 17.4 years) captured in another clinical study (13).

### Structural requirements included

- Sufficient reliability and compliance with treatment-related requirements both on the parents’ and patient’s sides.
- The ability of the parents to independently handle the assist device with respect to changes in the pump system, control of anticoagulation treatment according to the manufacturer’s instructions, recognition of possible medical emergency situations and the necessary actions to take as well as the possible independent changing of dressing materials.

### Organizational requirements focused on

- The design of a strict follow-up plan with respect to professional medical and technical checks.
- Creating capacities for regular interdisciplinary outpatient check-ups at the clinic (including consultation of pediatric cardiologists, pediatric cardiac surgeons, perfusionists, nursing staff, and physiotherapists).
- The presence of emergency structures nearby patients’ home.
- Having sufficient knowledge about required emergency procedures on the parents’, patients’ and potentially involved professionals’ sides.
- The possibility of refunding the cost of the treatment.

## Results

### Medical aspects

In the last 2 years we were able to identify a total of three EXCOR^®^ VAD patients (aged 10 months, 20 months, and 17 years, see anthropometric data, Table 1) at the Department of Pediatric Cardiology, University Hospital Erlangen, who met the inclusion criteria for VAD treatment supported with the EXCOR^®^ Active driving unit. All three patients were being treated with a paracorporeal ventricular assist device connected to a paracorporeal blood pump with a size lower than 60 ml and presented with stable laboratory parameters (hemogram, kidney and liver function, coagulation). Anticoagulation therapy during VAD treatment was given according to the manufacturer’s instructions for treatment with paracorporeal ventricular assist devices (Supplementary Table 3, EXCOR^®^, Berlin Heart GmbH) using vitamin K antagonists and platelet inhibitors such as acetylsalicylic acid or Dipyridamole (10, 14). None of the patients were suffering from a wound infection or wound dehiscence. All three patients had stable echocardiographic findings before their switch to EXCOR^®^ Active with respect to ventricular function, ventricular filling, the presence of intracavitary thrombi and valvular insufficiencies (Supplementary Table 1).

**TABLE 1:** Anthropometric characteristics of the patients at different stages of therapy.

Anthropometric data	Admission	Pre EXCOR	Pre EXCOR active	Post EXCOR active	Most recent data
**Patient 1**					
Weight (kg) (percentile/z-score)	3.49 (6/−1.55)	4.45 (<1/−4.1)	9.2 (46/−0.10)	9.09 (31/−0.51)	11.2 (28/−0.59)
Height (cm) (percentile/z-score)	52 (9/−1.37)	56 (<1/−5.01)	n.a.	n.a.	80 (4/−1.78)
HR (per min)	173	164	146	121	111
Blood pressure (mmHg)	66/36 (48)	n.a.	97/51 (69)	97/52 (68)	87/60 (72)
**Patient 2**					
Weight (kg) (percentile/z-score)	7.03 (16/−0.98)	8.54 (21/−0.82)	n.a.	11.4 (8/−1.39)	15.5 (50/−0.01)
Height (cm) (percentile/z-score)	69 (54/+0.1)	n.a.	n.a.	n.a.	98 (31/−0.51)
HR (per min)	121	110	122	97	85
Blood pressure (mmHg)	70/44 (52)	95/63 (80)	n.a.	91/66 (78)	105/58 (84)
**Patient 3**					
Weight (kg) (percentile/z-score)	49.1 (9/−1.35)	n.a.	42.5	43.4	43.5
Height (cm) (percentile/z-score)	160 (11/−1.23)	n.a.	160	160	160
HR (per min)	79	111	76	79	64
Blood pressure (mmHg)	114/80 (93)	115/86 (74)	−74	104/57 (73)	96/57 (69)

For all three patients, the switch to EXCOR^®^ Active was performed in the patient’s room in the presence of an interdisciplinary team consisting of pediatric cardiologists and perfusionists specialized in pediatric patients. Supervising was made by echocardiographic diagnostic before and after the switch (ventricular function, ventricular filling and valvular insufficiencies).

**FIGURE 1:**
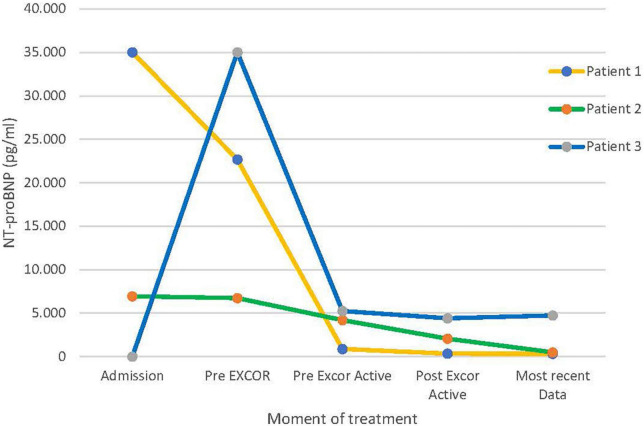
Levels of NT-proBNP over the course of the treatment.

The clinical condition of all three patients remained unchanged after the switch to EXCOR^®^ Active. Echocardiographic findings and laboratory controls, especially NT-proBNP, used as a marker of cardiac insufficiency (15), remained stable while on EXCOR^®^ Active (Figure 1 and Supplementary Table 1).

The motor development of all three patients was assessed by regular physiotherapeutic evaluations while in hospital. Due to the initially critical condition of all three patients and the huge differences between them with respect to their ages and individual courses of their disease, this study does not allow for standardized results of motor development for the in-hospital period. After becoming stable on the EXCOR^®^ Active, all three patients underwent detailed and age-adapted physiotherapeutic evaluations. All three patients underwent positive motor development, with results entering the normal range of the MRC muscle scale (16) for patient 3 and almost age-appropriate psychomotor development in the two younger patients.

**FIGURE 2:**
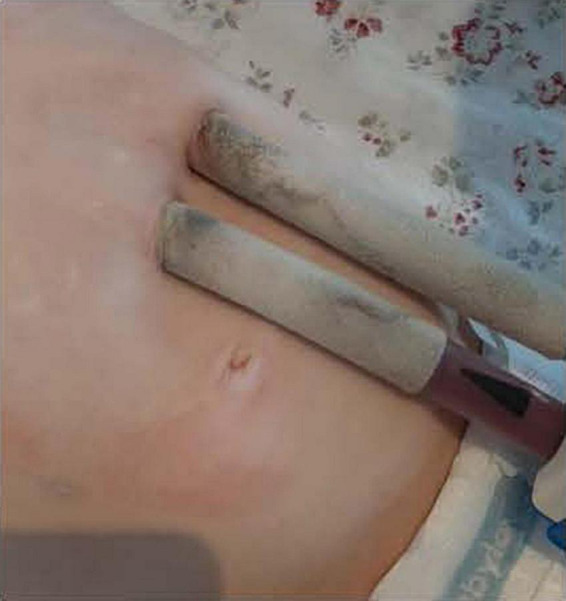
No detectable wound healing disorders after 5 months out of hospital.

For all three patients, after discharge home, the wounds at the cannula sites showed no signs of infection or dehiscence, comparing favorably with wounds of patients in hospital (Figure 2).

### Technical aspects

**FIGURE 3:**
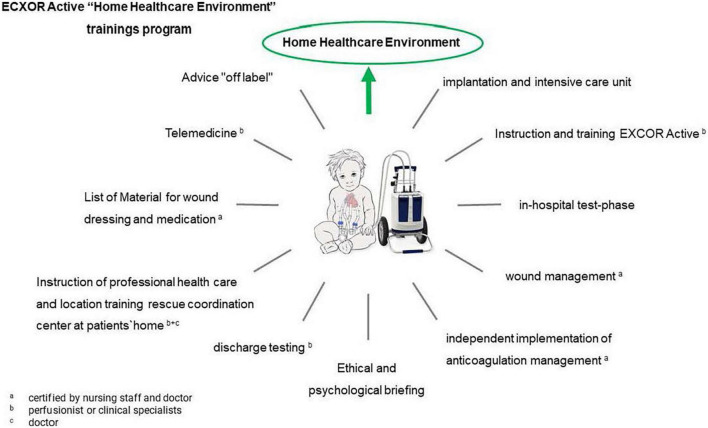
“Clock of action” until official hospital discharge with EXCOR^®^ active.

In all three patients, the switch of the driving unit from IKUS to EXCOR^®^ Active was performed without any technical problems. It was performed by specialized perfusionists and supervised by pediatric cardiologists who were guided by the patient’s echocardiogram. The EXCOR^®^ Active driving unit was preset at the same VAD pump frequency, the driving tubes were placed from the IKUS onto the EXCOR^®^ Active and fine technical adjustments were carried out during the first 15 min after the switch with the help of real-time echocardiographic monitoring, capturing slight changes in valvular competency and the filling and function of the native heart. For an easy and safe handling of the EXCOR^®^ Active, the alarm threshold was set at 60% of the current cardiac output. This was in order to avoid false-negative alarms due to normal movements and slight bending of the cannulas. Filling and emptying pressures of all three devices were in the recommended and expected operation ranges. To guarantee a safe stay at home, remote medical support with data-secure exchange of patient-related data and videoconferencing was implemented. The only hardware necessary for the parents was an electronic device such as a smart phone, tablet or laptop equipped with a camera and a microphone. Communication on the hospital’s side was easy due to our pre-existing telemedical communication service (16). The installation of the required software (HealthDataSpace© or Cisco webex©) and training during the hospital stay and after home discharge was supported by the telemedical team at the Department of Pediatric Cardiology, Erlangen. Regular videoconferences were held at least once a week according to our follow-up plan (Figure 3).

### Ethical aspects

Possible risks for patients consisted of: bleeding and thromboembolic complications caused by inappropriate anticoagulation therapy leading to a cerebral insult or acute heart failure; wound infection leading to readmission and additional surgical interventions; acute disconnection or damage to the EXCOR^®^ Active, resulting in an emergency or sudden death. Further possible issues included the possible stigmatization of the patient while in contact with their environment. The negative psychological effects on the parents caused by the heavy burden of new responsibilities had to be taken into consideration. Possible benefits included a positive influence on patients’ emotional, social and motor development due to a being in a familiar environment, normalized social contacts and activities and reduced exposure to multi-drug-resistant hospital infections. Possible benefits concerning the parents included improved self-confidence, a simplified daily routine, new possibilities for concomitant employment and psychological improvement due to having a more normal social life. Individual ethical considerations with regard to a gain in autonomy vs. potential risks while in HHE have been discussed individually among patients (if possible), parents, medical professionals, psychologists and, as in our first case, a committee from the ethics department of the University of Erlangen. All three study patients could subsequently be enrolled and were closely supported by a psychological team.

### Psychological aspects

The quality of life (QoL) of two individual patients (this study) in HHE was compared to the QoL of pediatric in-hospital patients on EXCOR^®^ Active (Miera et al) by means of PedsQL scores (Table 2). The summary statistics are shown in Table 2; the PedsQL scores are shown as mean (± SD). PedsQL-data from HHE-patients was obtained in 03-04/2022, detailed results of serial dates of questioning are displayed in Supplementary Table 4.

**TABLE 2:** Quality of life data of 2 of the discharged patients in comparison to 11 in-hospital patients with EXCOR^®^ VAD on EXCOR^®^ active: HRQL, health-related quality of life; N, number of patients in the analysis set; n, number of patients with evaluable data; SD, standard deviation.

	In-hospital (*N* = 11)		Out-of-hospital (*N* = 2)		Absolute change in summary	
			
	*n*	Mean (± SD)	Patient 2	Patient 3	Mean patient 2	Mean patient 3
Total score	11	67 (21)	52	72	−15	5
Parent HRQL summary score	11	70 (24)	44	79	−26	9
Family functioning summary	11	65 (19)	44	78	−21	13
Physical functioning score	11	63 (27)	50	50	−13	−13
Emotional functioning score	11	71 (26)	45	100	−26	29
Social functioning score	11	68 (29)	44	69	−24	1
Cognitive functioning score	11	76 (21)	35	100	−41	24
Communication score	11	72 (21)	83	75	11	3
Worry score	11	55 (25)	80	30	25	−25
Daily activities score	9	49 (23)	25	50	−24	1
Family relationships score	11	72 (17)	55	95	−17	23

For patient 3, there were marked positive differences, most notably in the emotional functioning score (100 vs. 71), cognitive functioning score (100 vs. 76), and family functioning score (95 vs. 72). In contrast, the most important negative difference was in the worry score (30 vs. 55).

For patient 2, the largest positive difference was observed in the worry score (80 vs. 55). In contrast, the largest negative difference was found in the cognitive functioning score (35 vs. 76).

### Structural aspects

A crucial criterion for a safe and stable home-treatment with EXCOR^®^ Active is being able to rely on full compliance with treatment on both the parents’ and patient’s sides. The corresponding psychological assessments were repeated regularly to ensure this. The process of hospital discharge was accompanied by regular psychological briefings. A special focus was placed on reliable feedback, adequate self-assessment and stable relationships between all involved family members. Exclusion criteria were defined as unreliability with respect to application of medication and necessary measurements of the International Normalized Ratio (INR) as well as insufficient trustworthiness and lack of communication toward the team.

Parents were taught to handle the EXCOR^®^ Active themselves by instruction about adequate filling and emptying of the blood pump as well as recognition and correct interpretation of different EXCOR^®^ Active alarms. Practical training included using the driving and replacement units in case of failure, EXCOR^®^ flow sensor cleaning and cable protection (EXCOR^®^ flow sensor cable wrap), teaching about the necessary monthly checks and charging of the EXCOR^®^ Active back-up driving unit’s internal battery. Parents were briefed for as long as necessary by our perfusion team about emergency situations such as accidental disconnection of the artificial blood pump (urgent cross-clamping of the EXCOR^®^ cannula), leakage of the driving tube (repair with adhesive tape) and handling of the EXCOR^®^ manual. Parents were taught to independently change EXCOR^®^ cannula wound dressings through on-site education during routine changes of wound dressing (Table 3). Responsibility for using the correct anticoagulation had already been handed over to the parents during the hospital stay in order to monitor it and check that it was being done correctly. Concerning the medication of acetylic acid and Dipyridamole, control of laboratory parameters and adaptation of anticoagulation therapy is performed during regular in-hospital check-ups. Measurements of the INR and consecutive adjustments to the medication of vitamin K antagonists are made by the parents or the patient. The frequency of these measurements depends on the steadiness of the levels of INR. In case of doubt, the Department of Pediatric Cardiology is consulted. Anticoagulation regimen is according to the manufacturer’s instructions (Supplementary Table 3) (14).

**TABLE 3:** Hands-on plan for establishing a home healthcare environment.

	Doctor	Nursing	Perfusionist
Instruction and training EXCOR^®^ Active			**x**
Clarification of legal aspects	**x**		
Cost coverage by health insurance	**x**		
In-hospital test-phase			**x**
Wound management (certificate)	**x**	**x**	
Independent implementation of coagulation management (certificate)	**x**	**x**	
Psychological briefing/ethical statement	**x**	**x**	**x**
Test trips out of hospital			**x**
Instruction of professional healthcare at home (flyer)	**x**		**x**
List of wound dressing		**x**	
List of medication	**x**		
Telemedical coaching	**x**		**x**
Medical recommendation for “out-of-hospital” use	**x**		
Informed consent “off label use”	**x**		

### Organizational requirements

**FIGURE 4:**
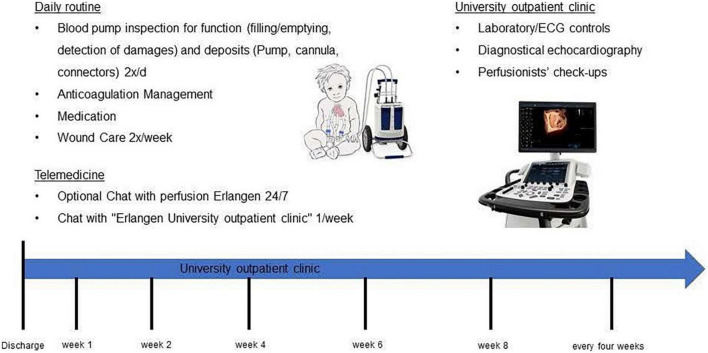
Follow-up plan after discharge.

The design of a strict follow-up plan with respect to professional check-ups was an essential part of this project and is displayed in detail in Figure 4.

Regular in-hospital checks included a technical check-up of the VAD by our perfusionists at each appointment at the university outpatient clinic in the Pediatric Cardiology Department in Erlangen as well as echocardiographic checks at least every 4 weeks.

Regular telemedical consultations were initially held individually and were established on a weekly basis. A detailed questionnaire for each telemedical consultation can be found in the (Supplementary Table 2).

The presence of emergency structures near the patients’ homes was checked before hospital discharge and the patients, parents and all potentially involved medical structures (ambulance services, pediatricians/pediatric cardiologists, children’s hospitals in the surrounding areas) were instructed about the necessary measures that would be required in case of an emergency and provided with a short, printed instruction and a remote briefing.

To standardize and simplify a project as complex as creating a healthcare environment for treating pediatric VAD patients, we created a “hands-on” plan of all the steps that have to be considered (Table 3).

Refunding the cost of VAD treatment was a crucial aspect of successful home discharge and was discussed on a case-by-case basis with the involved insurance companies. A positive outcome was achieved in every case. Legal aspects had been discussed previously with the hospital’s regulatory department and addressed with a written informed consent for off-label-use of the EXCOR^®^ VAD in an HHE.

## Specific case presentations

**FIGURE 5:**
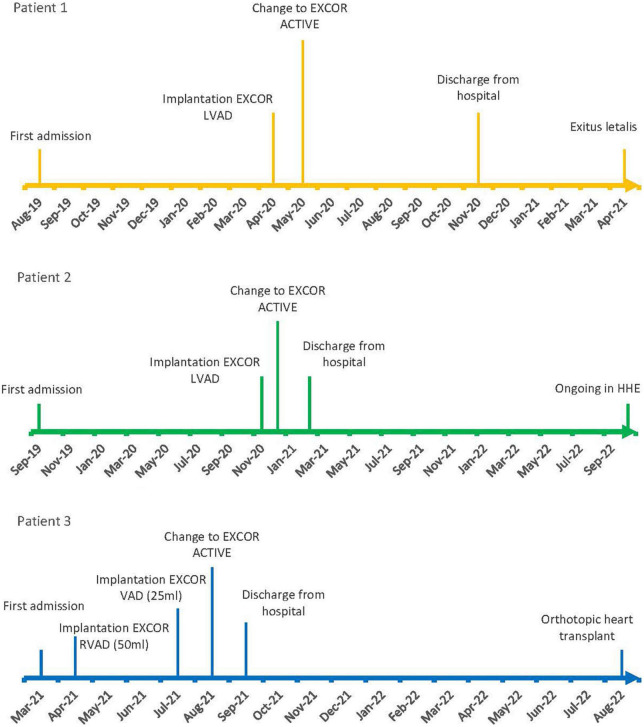
Milestones of treatment.

Here we present the first three cases of the use of an HHE in pediatric patients supported by an EXCOR^®^ Active driving unit worldwide. These cases provide a guideline for the safe home discharge of pediatric patients being treated with a VAD. Anthropometric characteristics of each patient can be taken from Table 1. A timeline of each patient’s individual treatment is displayed in Figure 5.

### First patient

The first patient was the fifth patient worldwide and youngest EXCOR^®^ patient supported with the EXCOR^®^ Active driving unit. Diagnosed at the age of 3 months, this little toddler had signs of severe heart insufficiency secondary to dilated cardiomyopathy. After 5 months of forced optimal medical treatment (OMT), there were no signs of improvement. Subsequently, at the age of only 8 months and with a total body weight of 8 kg, an emergency implantation of a 15 ml EXCOR^®^ blood pump with an IKUS driving unit had to be performed. In May 2020, after 2 months of stable VAD therapy with the IKUS driving unit, this was the smallest patient to be switched onto the EXCOR^®^ Active worldwide.

Due to the COVID-19 pandemic, hospital visits and possible intra-hospital mobility were very restricted and were only allowed for one parent at a time. Subsequently, this little patient suffered from severe psychomotor retardation. Despite the efforts of both the parents and hospital team, no official legal permit for home discharge was given. Still, all other defined requirements could be fulfilled (Table 2). Being clearly informed about the off-label use and considering the ethical framework consisting of beneficence, non-maleficence, autonomy and justice (17) in an ethics counseling (Ethics Committee, University Hospital Erlangen), the parents gave their informed consent for HHE therapy. In November 2020, the first pediatric patient being treated with a 15 ml LVAD and an EXCOR^®^ Active driving unit worldwide could be successfully discharged home.

No significant problems occurred during the first 5 months at home and the little patient showed an impressive improvement in his psychomotor development. Regular checks, including tele-medical video consultations, were performed according to the follow-up treatment plan.

Unfortunately, in April 2021, the patient developed a massive intracerebral hemorrhage during a routine hospital stay for a planned switch of the blood pump and subsequently died.

### Second patient

In November 2020, a second patient was selected for EXCOR^®^ Active support. This now 3-year-old boy, who at the age of 20 months underwent an implantation of a 15 ml paracorporeal LVAD due to an acute cardiac decompensation on a non-compaction cardiomyopathy. Following the standard implantation procedure, the LVAD first was supported by an IKUS driving unit. In the course of the hospital stay, COVID-19-related restrictions impeded regular social contacts and strictly limited the little patient’s range of mobility. Consistently positive experiences with EXCOR^®^ Active in a HHE at this time allowed for a switch onto EXCOR^®^ Active in December 2020, followed by a successful home discharge in February 2021. Until now, this patient has the had the longest duration of EXCOR^®^ Active support in an HHE worldwide. The patient’s psychomotor development appears almost normal. A further aspect of special interest is wound management, which has been done without any complications at all. Regular weekly video conferences are held to follow the patient up and keep hospital checks to a minimum, with ambulatory visits every 4 weeks.

### Third patient

The third case is of a 17-year old female patient who had long-term VAD treatment for isolated failure of the right ventricle, which is a very rare pediatric pathology (18). Acute post-surgical complications after an elective patch-repair of a stenotic aortic arch (04/2021) led to cardiac resuscitation, mass-transfusion, emergency surgical repair and implantation of a VA-ECMO due to low-cardiac output. In the course of treatment, the patient suffered from isolated RV-failure due to damage after cardiac resuscitation, pronounced respiratory failure and pulmonary edema. The implantation of a temporary right ventricular assist device (RVAD) with an oxygenator was followed by the implantation of a durable paracorporeal RVAD (EXCOR^®^ 50 ml blood pump) combined with an iLA-active membrane lung (Fresenius Medical Care, Germany). As respiratory function improved with therapy, the iLA system was explanted. Only hours after explant, the patient developed acute pulmonary decompensation. Reduction of RVAD support to 36% (50 bpm, ca. 2.5 L/min.) improved the respiratory function. An interesting finding was that the oxygenator had a high resistance, concealing left ventricular overload. After 3 days, the RVAD blood pump was downsized in an off-label use from a 50 ml to a 25 ml blood pump (12 mm cannula to 9 mm pump connector) in 07/2021. A reduction in respiratory support could be achieved, with an RVAD flow of 2.1 L/min. The permanent use of the EXCOR^®^ Active driving unit became possible in 08/2021 and mobilization contributed to physical recovery of the patient as well as a successful reduction in long-term respirator dependency. After 175 days in hospital, the patient was discharged home successfully with an EXCOR^®^ 25 ml RVAD blood pump on EXCOR^®^ Active and recently underwent an orthotopic heart transplant (08/2022).

## Discussion

This globally unique project, aiming to establish a safe home health environment for the treatment of pediatric patients with a paracorporeal ventricular assist device, was a great challenge for all participants. Most importantly, no technical issues occurred in the HHE. The exchange of patients from the IKUS onto the EXCOR^®^ Active driving unit was a short procedure that could be performed safely and reliably. An equally safe setting can only be ensured in a specialized heart center with well-trained and experienced medical staff. Cardiac support on an EXCOR^®^ VAD with EXCOR^®^ Active as its driving unit allows for activity levels to be normalized as much as possible and can already help to improve patients’ physical health and quality of life during their hospital stay (13). Nevertheless, long-term hospitalization and long waiting times can lead to several problems, such as wound infections or systemic hospital acquired infections (19), possible delays in motor development, behavioral and psychological difficulties (20) and social deprivation in patients and parents (21). Therefore, discharge to a HHE as soon as possible seems to be crucial for these patients and their families (22).

In our experience, an HHE solution for pediatric patients on EXCOR^®^ VAD is feasible and safe. The effort required to establish an HHE program is high and takes both human resources and time for the appropriate mandatory training. Nevertheless, time should be taken, as comprehensive education has been proven to be of essential importance for the discharge of pediatric patients with complex needs (23). It is important to involve all professional parties (cardiologists, perfusionists, surgeons, nurses, psychologists as well as all caregivers, patients, and parents) at an early stage. It is essential to assess the individual needs of each patient with respect to the medical supervision required, the personal abilities of patients and parents and the subsequent training required. Moreover, it takes time to fulfill all requirements from health insurance companies and to investigate and instruct the professional healthcare network in case of emergency. Clearance by health insurance companies and full cost coverage are indispensable. Hands-on guides, standardized certifications, a strict follow-up-plan and the appropriate remote medical and technical support will simplify HHE treatment in this special patient group.

Through our three different cases, experience was gained of the advantages for different age groups and family settings and their special needs. Given the limited numbers, we can only discuss the individual HQRL-data of two HHE-patients in comparison to data of 11 pediatric in-hospital patients on EXCOR^®^ Active. Still, in the overall score, we did not notice a relevant difference between the in-hospital group and the two patients in HHE. The opposite developments of the worry scores and the cognitive functioning scores of the two patients in HHE underline the influence, different HHE settings and patient specific circumstances can have on this new regimen. Therefore, an individual character of this treatment option will remain for each patient.

Schweiger et al. demonstrated that adolescents benefit from normalized social contacts and activities, including normal school attendance, even when they have an intracorporeal left ventricular assist device (24). But this only works if patients do not suffer from depression due to their mechanical life support (25) and pull back from their social surroundings, as was reported with the third patient. In this case, only intense psychological support can help to stabilize the patient’s mental state. Like in the second case, for parents with more children than only the affected patient, the additional workload can be stressful. This might also interfere with their capacity to participate in regular work life. Early support from social workers and close psychological support will help to avoid long-term problems like anxiety and depression (26) or loss of employment. Regular and high-quality telemedical support is essential to reduce the number of ambulatory visits and additional costs. Unfortunately, the financial recompense by health insurance companies for video-consultations is still an open topic to be negotiated in the future. As in the first case, parents with only one child as the patient have a tremendous opportunity to more easily pursue their work, enjoy a normalized social life, and take full advantage of the HHE concept like for the children on LVAD treatment referred to by Schweiger et al. (24).

As EXCOR^®^ VAD is intended to be used as bridge to transplant or bridge to recovery, the newly developed HHE concept offers the possibility of reducing the number of adverse events (e.g., wound infection) and allows for increased mobility with the possibility of improved psychomotor development and a social life, analogous to former experiences with HHE in pediatric patients with special health care needs (27).

With respect to the medical contemplation of this project, the economic implications of this project were not analyzed. Nevertheless, there are indications that it may allow for reductions in cost to the public health system with the simultaneous conservation of scarce hospital resources.

Future analysis will have to include more patients and a standardized examination of their motor development during their hospital stay and after home discharge. Legal barriers will have to be overcome and the investigation of the financial aspects will be crucial as proper reimbursement is an integral part of an HHE solution.

### Limitations

As this is the first study of an off-label HHE solution to pediatric EXCOR^®^ VAD-support with the mobile driving unit EXCOR^®^ Active worldwide, only a partially retrospective and single-center analysis of a limited number of patients can be reported. Still, this study was the first iteration of a possibly revolutionary concept in the long-term treatment of pediatric patients on VAD support and has been designed as a feasibility study with a focus on patient safety.

Furthermore, an important limiting factor is the patient’s domestic environment, with compliance on both family’s and patients’ sides, an appropriate technical and medical setting and the willingness of all involved participants being absolutely essential.

## Conclusion

Discharge from hospital for pediatric patients on current paracorporeal ventricular assist support is feasible and can be managed safely by utilizing the mobile driving unit EXCOR^®^ Active. The switch from the stationary driving unit as well as the preparations for discharge have to be supervised by an experienced interdisciplinary team of pediatric cardiologists, surgeons, specialized perfusionists and nurses. Proper instruction, sufficient personal compliance and resources on the patients’ and families’ sides are essential. The availability of professional healthcare nearby the domestic environment and adequate briefings about what to do in case of emergency are crucial. Telemedical infrastructure can simplify follow up. Until now, this HHE solution is an off-label use, requiring individual ethical consent for each case. Nevertheless, full cost coverage by the responsible health insurance companies is indispensable for a successful project, that can help to improve patients’ psychomotor development, offer a normalized social life and strengthen both patients’ and parents’ resources. Legal approval will help to gain a broader experience as more patients can be included in further studies, which could focus even more on patients’ psychomotor development and the psychological aspects of a VAD-therapy in an HHE.

## Data availability statement

The original contributions presented in this study are included in the article/Supplementary material, further inquiries can be directed to the corresponding author.

## Ethics statement

The studies involving human participants were reviewed and approved by the Department of Clinical Ethics, Ethics Committee, University Hospital Erlangen. Written informed consent to participate in this study was provided by the participants’ legal guardian/next of kin. Written informed consent was obtained from the individual(s), and minor(s)’ legal guardian/next of kin, for the publication of any potentially identifiable images or data included in this article.

## Author contributions

KR, FM, and SD conceived and designed the study. KR, FM, AT, NK, SB, and AP acquired the data. KR, FM, SD, and OD analyzed the data, interpreted the data, and drafted the manuscript. All authors critically reviewed the manuscript and are fully accountable for all aspects of work, ensuring integrity, and accuracy.

## Abbreviations

BVAD: biventricular assist device
ECG: electrocardiogram
HHE: home healthcare environment
HQRL: health-related quality of life
HR: heart rate
INR: international normalized ratio
NT-proBNP: N-terminal prohormone of brain natriuretic peptide
LVAD: left ventricular assist device
OMT: optimal medical treatment
PedsQL: pediatric quality of life inventory
RVAD: right ventricular assist device
VAD: ventricular assist device.
