# Supplementary material for: Mobility and freedom of movement: A novel out-of-hospital treatment for pediatric patients with terminal cardiac insufficiency and a ventricular assist device
Source: Front Cardiovasc Med. 2022 Nov 16;9:1055228. doi: 10.3389/fcvm.2022.1055228 (PMC9708718; doi:10.3389/fcvm.2022.1055228)
Supplement: Supplementary file 1 [file Table_1.pdf]

Supplementary material

Table 1 suppl.: Important findings in echocardiography/ECG and exemplary laboratory values during the different stages of treatment

	Admission	Pre EXCOR	Pre EXCOR Active	Post EXCOR Active	Most recent data
**Patient 1**					
**Echocardiography**					
**EF (%)**	39	21	29	33	34
**TI (mmHg)**	n.a.	10	25	n.a.	n.a.
**E`lat (m/s)**	0,06	n.a.	n.a.	0,04	n.a.
**E`septal (m/s)**	0,17	n.a.	n.a.	0,05	n.a.
**PVmax (m/s)**	0,88	1,11	1,16	1,15	1,18
**AV VTI (m/s)**	6,1	11,5	23	22	n.a.
**MAPSE (cm)**	3,6	4,6	n.a.	5,1	n.a.
**TAPSE (cm)**	8,9	n.a.	n.a.	10,6	n.a.
**Electrocardiogram**					
**HR (per min)**	173	164	146	121	111
**cardiac axis (°)**	111°	122°	138°	112°	130°
**QRS (ms)**	60	87	83	81	83
**P (ms)**	59	44	106	84	107
**PQ (ms)**	111	90	149	155	174
**QTc (ms)**	337	394	438	376	427
**Laboratory values**					
**NT-proBNP (pg/ml)**	>35.000	22.679	872	336	312
**GOT (U/l)**	39	42	40	39	43
**GPT (U/l)**	11	24	16	22	24
**GGT (U/l)**	n.a.	n.a.	n.a.	n.a.	18
**Patient 2**					
**Echocardiography**					
**EF (%)**	32	30	n.a.	39	52
**TI (mmHg)**	18	18	44	20	18
**E`lat (m/s)**	0,07	0,02	n.a.	0,09	0,11
**E`septal (m/s)**	0,05	0,06	n.a.	0,05	0,04
**PVmax (m/s)**	0,89	0,99	n.a.	0,97	0,84
**AV VTI (m/s)**	18,7	17,7	n.a.	15	n.a.
**MAPSE (cm)**	5,4	5	6,8	7,7	9,3
**TAPSE (cm)**	17	13,7	7,8	7,3	17
**Electrocardiogram**					
**HR (per min)**	121	110	122	97	85
**cardiac axis (°)**	90°	76°	86°	79°	84°

**QRS (ms)**	88	86	75	89	72
**P (ms)**	53	98	74	99	80
**PQ (ms)**	89	134	116	137	130
**QTc (ms)**	431	414	414	439	390
**Laboratory values**					
**NT-proBNP (pg/ml)**	6942	6.733	4200	2066	490
**GOT (U/l)**	50	67	57	46	43
**GPT (U/l)**	14	n.a.	14	8	8
**GGT (U/l)**	n.a.	n.a.	69	27	12
**Patient 3**					
**Echocardiography**					
**EF (%)**	86	36	40	58	44
**TI (mmHg)**	none	21	28	27	23
**E`lat (m/s)**	n.a.	0,05	n.a.	0,09	0,1
**E`septal (m/s)**	n.a.	0,03	n.a.	0,06	0,06
**PVmax (m/s)**	1,07	n.a.	0,3	0,87	1,28
**AV VTI (m/s)**	27	12,9	15,8	16	20
**MAPSE (cm)**	15	7,8	n.a.	7	9
**TAPSE (cm)**	22	1	n.a.	n.a.	5
**Electrocardiogram**					
**HR (per min)**	79	111	76	79	64
**cardiac axis (°)**	76°	106°	78°	87°	105°
**QRS (ms)**	90	133	94	78	101
**P (ms)**	105	none	74	80	71
**PQ (ms)**	126	none	205	196	207
**QTc (ms)**	393	439	426	429	464
**Laboratory values**					
**NT-proBNP (pg/ml)**	n.a.	> 35.000	5232	4400	4728
**GOT (U/l)**	22	760	16	16	20
**GPT (U/l)**	14	685	12	9	16
**GGT (U/l)**	12	193	47	33	32
