# Supplementary material for: Mobility and freedom of movement: A novel out-of-hospital treatment for pediatric patients with terminal cardiac insufficiency and a ventricular assist device
Source: Front Cardiovasc Med. 2022 Nov 16;9:1055228. doi: 10.3389/fcvm.2022.1055228 (PMC9708718; doi:10.3389/fcvm.2022.1055228)
Supplement: Supplementary file 2 [file Table_2.pdf]

Supplementary material

Table 2 suppl.: Questionnaire for remote consultations for VAD-patients in a home healthcare environment

Patient:___________________________ Date: ____________________		Doctor:_______________________	
**Question**	**Answer**	**Consequence**	**Notes**
How are you today?			
How ist he patient?			
Does your child feel weak or limitid in its power?			
What kind of activities could you perform dring the last week?			
Have there been any relevant changes since the last consultation?			
How many alarms did occur since the last consultation?			
Have the alarms be reasonable?			
Have there been any technical issues?			
How does the INR count (today/in the course of the last week)?			
Which is the actual medication of the patient?			
Has there been occurrence of epistaxis?			
Did you register changes in the blood pump (filling/emptying/thrombus)?			
How is the setting (driving unit/pressure/frequency)?			
When is your next ambulatory visit?			
Do you have any more questions?			
