# Supplementary material for: Mobility and freedom of movement: A novel out-of-hospital treatment for pediatric patients with terminal cardiac insufficiency and a ventricular assist device
Source: Front Cardiovasc Med. 2022 Nov 16;9:1055228. doi: 10.3389/fcvm.2022.1055228 (PMC9708718; doi:10.3389/fcvm.2022.1055228)
Supplement: Supplementary file 3 [file Table_3.pdf]

EXCOR® Pediatric

Anticoagulation Guideline Summary

Medications	Dosing Guidelines	Labs and Target Values
**Unfractionated Heparin (UFH)**<br>Start when:<br>• > 24 hours post-op (48 hours if ECMO)<br>• no bleeding (esp. infants and young kids) and platelets > 20 k/µl)<br>• normal coag. status (TEG and plt. agg.)	Start with:<br>Age < 12 mo.<br>- 15 IU/kg/hr (no bolus)<br>- after 6 hours, ↑ to 28 IU/kg/hr<br>Age > 12 mo.<br>- 10 IU/kg/hr (no bolus)<br>- after 6 hours, ↑ to 20 IU/kg/hr	aPTT – check every 6 hours or after dose change<br>**Targets**<br>– PTT - 1,5 – 2,5 times the normal<br>– UFH/Anti-FactorXa level – 0,35 – 0,5 IU/ml
**Low Molecular Weight Heparin (LMWH)**<br>Start when creatinine normal and no bleeding or if<br>• unable to tolerate PO<br>• unstable INR's<br>• convert from UFH after 48 hours if stable and no bleeding	**Initial Dosing: eg. Enoxaparin**<br>Age < 3 mo. 1.8 mg/kg 2x/d<br>Age 3-12 mo. 1.4 mg/kg 2x/d<br>Age 1-5 yrs. 1.2 mg/kg 2x/d<br>Age 6-18 yrs. 1.1 mg/kg 2x/d<br>**If low INR with Vitamin K antagonist:**<br>INR = 2.0 – 2.7<br>- prophylactic dose (1.0 mg/kg/d)<br>INR < 2.0 (or use IV UFH)<br>- therapeutic dose (1.0 mg/kg 2x/d)	**Target: Anti-Factor Xa level 0,6 – 1,0 IU/ml**<br>– draw level 4 hours after 2nd dose or dose change until stable, then twice a week.<br>– Discontinue UFH and start LMWH simultaneously
**Vitamin K Antagonist (can bridge with LMWH)**<br>Start if age > 12 mo. AND if<br>• enteral feeding possible hemodynamically stable	**Initial Dosing: eg. Warfarin**<br>- 0.2 mg/kg/d (Maximum 5 mg/d)	– Target INR 2.7-3.5<br>Change to LMWH if unstable INR's
**Platelet Inhibitors**<br>Start when platelets >40 k/µl<br>Dipyridamole – POD 2 and ADP > 50 %<br>Aspirin – POD 4, drains out and ARA > 50 % or POD 7 if drains still in	Start with:<br>- 4 mg/kg/d in 4 divided doses (Max dose 15 mg/kg/d)<br>- 1 mg/kg/day	**Targets: Platelet aggregation**<br>– ADP –activity < 50 %<br>– ARA –activity < 30 %<br>– Collagen acts as a control and should not be suppressed. Epinephrine target 40-50 %

Sources: EXCOR® VAD with Stationary Driving Unit Ikus – Instructions for Clinical Use
EXCOR® Pediatric IDE Study Protocol, EXCOR® IKUS Canadian Manual

* This is only a guideline and does not substitute clinical evaluation and judgement of each individual patient. This guideline does not factor in the usage of thromboelastrography.
