# Supplementary material for: Mobility and freedom of movement: A novel out-of-hospital treatment for pediatric patients with terminal cardiac insufficiency and a ventricular assist device
Source: Front Cardiovasc Med. 2022 Nov 16;9:1055228. doi: 10.3389/fcvm.2022.1055228 (PMC9708718; doi:10.3389/fcvm.2022.1055228)
Supplement: Supplementary file 4 [file Table_4.pdf]

Supplemetary material

Table 4 suppl.: Detailed serial PedsQL of patient 2 and patient 3 (obtained between March and April 2022).

	Patient 3						Patient 2					
	08.03. - 13.03.	14.03. - 20.03.	21.03. - 27.03.	28.03. - 03.04.	Mean	Mean total	03.03.	10.03.	17.03.	24.03.	Mean	Mean total
**Physical Functioning (*problems with…)***						50						50
1. I feel tired during the day	25	25	25	25	**25**		50	50	25	25	**38**	
2. I feel tired when I wake up in the morning	25	25	25	25	**25**		50	50	50	25	**44**	
3. I feel too tired to do the things I like to do	50	50	50	50	**50**		50	50	50	25	**44**	
4. I get headaches	50	50	50	50	**50**		75	50	50	0	**44**	
5. I feel physically weak	50	50	50	50	**50**		50	50	25	0	**31**	
6. I feel sick to my stomach	100	100	100	100	**100**		100	100	100	100	**100**	
**Emotional Functioning (*problems with…)***						100						45
1. I feel anxious	100	100	100	100	**100**		0	50	50	25	**31**	
2. I feel sad	100	100	100	100	**100**		50	25	25	25	**31**	
3. I feel angry	100	100	100	100	**100**		75	75	75	75	**75**	
4. I feel frustrated	100	100	100	100	**100**		50	50	50	25	**44**	
5. I feel helpless or hopeless	100	100	100	100	**100**		50	50	50	25	**44**	
**Social Functioning (*problems with…)***						69						44
1. I feel isolated from others	75	75	75	75	**75**		25	50	50	50	**44**	
2. I have trouble getting support from others	100	100	100	100	**100**		75	75	75	75	**75**	
3. It is hard to find time for social activities	50	50	50	50	**50**		50	75	25	0	**38**	
4. I do not have enough energy for social activities	50	50	50	50	**50**		25	25	0	25	**19**	
**Cognitive Functioning (*problems with…)***						100						35
1. It is hard for me to keep my attention on things	100	100	100	100	**100**		25	50	25	25	**31**	

2. It is hard for me to remember what people tell me	100	100	100	100	**100**		25	25	25	25	**25**	
3. It is hard for me to remember what I just heard	100	100	100	100	**100**		25	25	25	25	**25**	
4. It is hard for me to think quickly	100	100	100	100	**100**		50	50	50	50	**50**	
5. I have trouble remembering what I was just thinking	100	100	100	100	**100**		50	25	50	50	**44**	
**Communication (*problems with…)***						**75**						**83**
1. I feel that others do not understand my family's situation	75	75	75	75	**75**		100	50	75	75	**75**	
2. It is hard for me to talk about my child's health with others	50	50	50	50	**50**		100	75	25	100	**75**	
3. It is hard for me to tell doctors and nurses how I feel	100	100	100	100	**100**		100	100	100	100	**100**	
**Worry (*problems with…)***						**30**						**80**
1. I worry about whether or not my child's medical treatments are working	50	50	50	50	**50**		100	100	100	50	**88**	
2. I worry about the side effects of my child's medications/medical treatments	25	25	25	25	**25**		100	100	100	25	**81**	
3. I worry about how others will react to my child's condition	50	50	50	50	**50**		100	100	100	100	**100**	
4. I worry about how my child's illness is affecting other family members	25	25	25	25	**25**		100	100	100	100	**100**	
5. I worry about my child's future	0	0	0	0	**0**		50	25	25	25	**31**	
**Daily Activities (*problems with…)***						**50**						**25**
1. Family activities taking more time and effort	25	25	25	25	**25**		0	25	25	25	**19**	
2. Difficulty finding time to finish household tasks	75	75	75	75	**75**		0	50	0	50	**25**	
3. Feeling too tired to finish household tasks	50	50	50	50	**50**		25	0	50	50	**31**	
**Family Relationships (*problems with…)***						**95**						**55**
1. Lack of communication between family members	100	100	100	100	**100**		50	25	25	75	**44**	
2. Conflicts between family members	100	100	100	100	**100**		25	25	75	75	**50**	
3. Difficulty making decisions together as a family	100	100	100	100	**100**		100	75	75	75	**81**	
4. Difficulty solving family problems together	100	100	100	100	**100**		50	50	50	75	**56**	
5. Stress or tension between family members	75	75	75	75	**75**		25	25	50	75	**44**	
